# Impact of post-COVID symptoms on activity and participation of women and men

**DOI:** 10.1038/s41598-024-74568-1

**Published:** 2024-10-17

**Authors:** Jana Rosenstein, Christina Lemhöfer, Dana Loudovici-Krug, Christian Sturm, Andrea Bökel

**Affiliations:** 1 Department of Rehabilitation- and Sports Medicine, Hannover Medical School, 30625 Hanover, Germany; 2 Institute of Physical and Rehabilitation Medicine, Jena University Hospital, 07747 Jena, Germany

**Keywords:** Infectious diseases, Diseases, Health care

## Abstract

Post-COVID syndrome is affecting many organ systems and arises as a major public health problem with millions of cases worldwide. The primary aim of this study is the analysis of health problems, activity limitations and participation restrictions (ALPR) of participants with post-COVID symptoms and the investigation of correlations between these elements to derive statements about the rehabilitation need, also depending on sex. A retrospective cohort study was performed to collect longitudinal data from January 2022 to January 2023 using the Covid-19 Rehabilitation Needs Questionnaire (RehabNeQ). Patients completed the questionnaire at the Department of Rehabilitation- and Sports Medicine at Hannover Medical School. The 1st assessment included 307 study participants, of whom 54 showed up for the 2nd, 7 for the 3rd and one for the 4th assessment. Study participants with post-COVID symptoms also experience ALPR. The results show no significant difference in symptom intensity in women and men, but in intensity of ALPR. We found many correlations of varying degrees between various factors with ALPR. We found frequent correlations between fatigue and several ALPR. While these correlations apply to both sexes, we also found different correlations in women and men, indicating the different rehabilitation need of women and men.

## Introduction

More than 3 years after the onset of the global COVID-19 pandemic, post-COVID syndrome has emerged as a major factor of corona-virus related burden of disease, effecting many organ systems^1^ and arise as a major public health problem as millions of people are affected worldwide^2^.

The British National Institute for Health and Care Excellence (NICE) distinguishes ongoing and post-COVID as follows: ongoing symptomatic COVID-19 are health problems that persist for 4 weeks after the acute phase of the disease. Post-COVID-19 syndrome refers to symptoms that are present more than 12 weeks after infection and cannot be explained otherwise^3^.

Davis et al. assume, that minimum 65 million people worldwide have post-COVID, the real number is suspected to be higher due to undocumented cases^4^. The incidence of 12.5% of infected people is based on a conservative estimation^5^.

Patients with post-COVID experience a variety of symptoms, physical as well as psychological in nature: The 10 most common are fatigue (47%), dyspnea (32%), myalgia (25%), joint pain (20%), headache (18%), cough (18%), chest pain (15%), abnormal smell (14%), abnormal taste (7%) and diarrhea (6%)^6^. Cognitive impairment, brain fog, trouble sleeping, and palpitations are other common symptoms^6^.

The following factors are associated with an increased risk of developing persistent symptoms after a SARS-CoV-2 infection: age, hospitalization at the onset of symptoms^7,8^, dyspnea in the early phase of the disease, irregular auscultation findings of the heart, lungs, or other organs^8^, female gender, high burden of disease during the acute phase of the disease and asthma as preexisting condition^7^.

The above-mentioned symptoms can have negative effects on activity and participation^9^ as well as on quality of life and the ability to work^6^ and to date no effective treatment is known. In addition to this unmet needs regarding acute intervention, it is estimated that 15–35% of affected people are in need for rehabilitative interventions^9^.

Addressing this need, NICE recommends a multidisciplinary rehabilitation, which should include physical, psychological, and psychiatric management^3^. To individualize the treatment, a personalized rehabilitation program should be created together with each patient. To support the rehabilitation patients should be empowered to self-manage their symptoms^3^.

The aim of this study is the analysis of health problems, activity limitations and participation restrictions (ALPR) among women and men with post-COVID symptoms and the investigation of correlations between these factors, using the Covid-19 Rehabilitation Needs Questionnaire (RehabNeQ). Statements about the need for rehabilitation – also depending on sex - can be derived from the results. An additional goal is the assessment and analysis of longitudinal post-COVID courses.

## Materials and methods

### Study design

In this retrospective cohort study longitudinal clinical routine data were used. These data are patient reported information in the RehabNeQ online questionnaire. Patients completed the questionnaire for post-COVID patients at the special post-COVID outpatient clinic of the Department of Rehabilitation- and Sports Medicine at Hannover Medical School. At each appointment during the observation period of one year, patients received a tablet at the registration desk to fill out the questionnaire by themselves. All patients of the special post-COVID outpatient clinic in the period from January 2022 until January 2023 were included in the study. This means this is a complete recruitment during the observation period. These patients are characterized by having received a diagnosis of post-COVID from their GP or another doctor and have subsequently been referred to physical and rehabilitative medicine. Patients showed up with a brought range of COVID-19 associated symptoms in the special post-COVID outpatient clinic and were included accordingly. No patient was excluded from the study. Due to the post-COVID symptoms, some patients might not be able to fill the questionnaire completely by themselves. If necessary, the patients received help from a medical assistant in completing the questionnaire in order to address the bias that particularly severely impaired patients could not participate in the study.

### Measure

The items of the COVID-19 Rehabilitation Need Questionnaire (RehabNeQ) comprise individual questions to illustrate the particularities of a SARS-CoV-2 infection. A total of 57 items are assigned to 7 main categories: time of infection, health problems caused by SARS-CoV-2, treatment, activity and participation, quality of life and general health, health service provisions as well as basic demographic information^10^. To determine the most often named health problems and limitations in activity and participation, relative frequencies of the individual symptoms/limitations at the various points in time were summed up and sorted in descending order. A 5-point Likert scale was used to assess the intensity of symptoms and activity and restriction in participation, ranging from 0 (no problem) to 4 (extreme problem).

Data collection was conducted via a linked website, which is operated by the software company ComplySoft. For the analysis, ComplySoft provided the digitally collected data.

### Statistical analysis

Data analysis included descriptive analysis such as frequency, mean or median (Mdn.), standard deviation (SD) and percentages to describe the research data.

The Shapiro-Wilk test was used to check for normal distribution. Non-parametric tests were used when ordinal and interval-scaled data were not normally distributed. Signed-rank test was calculated to analyze significant differences in the frequencies of health problems and limitations in activity and participation at different times of (re-) assessment, Mann-Whitney U test was also performed to show significant differences in the above mentioned variables in women and men^11^. To examine the correlation of each symptom with each ALPR Spearman´s rank correlation was calculated^11^. The effect sizes are based on Cohen who proposed the following limits: a weak effect from *r* = 0.10, a medium effect from *r* = 0.30 and a strong effect from *r* = 0.50^12^.

All data were entered in IBM SPSS Statistics Version 28.0. A p-value less than 0.05 was considered significant.

The study was conducted in accordance with the Declaration of Helsinki and approved by the Ethics Committee of Hannover Medical School (No. 10874_BO_K_2023 on April 18th 2023). The Ethics Committee was represented by its Chairman Prof. Dr. Bernhard Schmidt. The need for informed consent was waived by the Ethics Committee of Hannover Medical School, since the data is used retrospectively and pseudonymized.

## Results

### Participants

In the special post-COVID outpatient clinic, 307 patients showed up during the recruitment period. Some, but not all patients showed up again and completed the assessment, which consists of the online questionnaire RehabNeQ, again. So we obtained data on 307 patients from the 1st assessment, of whom 54 (17.5%) showed up for the 2nd, 7 (2.3%) for the 3rd, and one (0,3%) person for the 4th assessment. Due to the high loss-to-follow-up and the small number of study participants in the 3rd and 4th assessments, we mainly report results from the first two assessments. More women than men presented in the special outpatient clinic. The mean age of the study participants was 45 years (SD 12.3) at the 1st assessment and 44 years (SD 11.3) at the 2nd assessment. Most of the patients (76.5%) showed up at the special outpatient clinic between 3 and 6 months after infection, while the total range is 3–24 months. Further sociodemographic characteristics of the study participants at different times of assessment are shown in Table 1.

**Table 1:** Sociodemographic characteristics of study participants at different times of assessment.

Characteristics of the participants	1st Assessment (*n* = 307)	2nd Assessment (*n* = 54)	3rd Assessment (*n* = 7)
Age at time of survey, years, mean (SD)	44.51 (12.3)	43.91 (11.3)	35.86 (8.4)
Sex, n (%)			
Male	88 (28.7)	16 (29.6)	1 (14.3)
Female	219 (71.3)	38 (70.4)	6 (85.7)
Size, cm, mean (SD)	172.5 (8.8)	171.7 (8.1)	171.0 (8.7)
Weight, kg, mean (SD)	79.8 (18.9)	80.56 (20.7)	83.7 (21.4)
Marital status, n (%)			
Single	73 (23.8)	13 (24.1)	1 (14.3)
Married	154 (50.2)	29 (53.7)	5 (71.4)
Cohabiting or in a partnership	51 (16.6)	8 (14.8)	1 (14.3)
Separated or divorced	26 (8.5)	4 (7.4)	0 (0.0)
Widowed	3 (1.0)	0 (0.0)	0 (0.0)

### Health problems, activity, and participation after COVID-19 infection

**Fig. 1:**
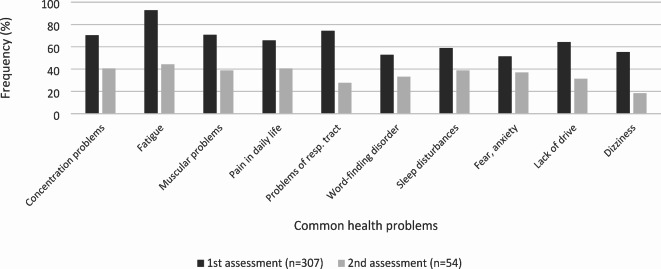
Frequencies of the most often reported symptoms of the 1st and 2nd assessment in %.

**Fig. 2:**
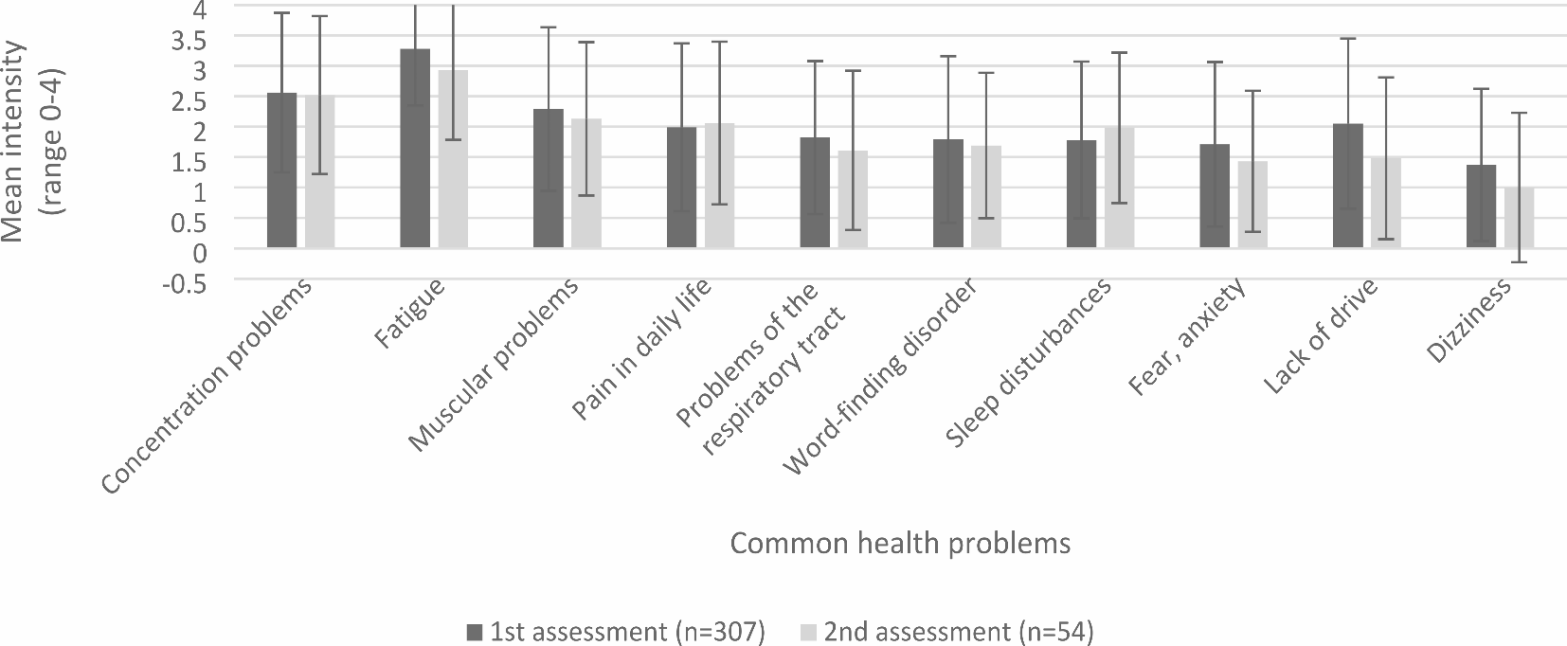
Intensity of the most common long-term symptoms over time including means and standard deviation.

In the online RehabNeQ questionnaire, the study participants indicated several health problems. It is noticeable that ‘fatigue’ is reported by 92.8% (*n* = 285) of study participants at the 1st assessment. Looking at the mean intensity of the health problems, ‘fatigue’ (mean = 3.3) and ‘concentration problems’ (mean = 2.6) were the symptoms with the highest intensity scores, with 4 being the highest available score meaning an extreme problem to rate intensity with. The frequencies of the most often reported health problems at the 1st and 2nd assessments are shown in Fig. 1. Figure 2 shows the mean intensity of the most common long-term symptoms over time, i.e. at different times of assessment. The range of reported intensity was 0–4.

**Fig. 3:**
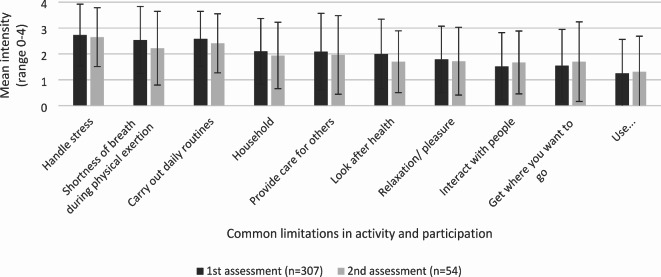
Mean intensity and standard deviation of the most mentioned limitations in activity and restriction in participation of the study participants at the various time points.

At the 1st assessment, 69.7% (*n* = 214) of the study participants stated that they were restricted in ‘carry out daily routines’ and that these restrictions had occurred after the infection with SARS-CoV-19. This means that ‘carry out daily routines’ is the item that was perceived as limited by most study participants at this time. The mean intensity of the most often mentioned ALPR of the study participants at the various time points are shown in Fig. 3. The three highest rated items on a scale from 0 to 4, at the 1st assessment are ‘handle stress’ (mean = 2.7), ‘carry out daily routines’ (mean = 2.6) and ‘shortness of breath during physical exertion’ (mean = 2.5).

Looking at the time course of patients who showed up again at the special outpatient clinic, the following stands out: The intensity of ‘fear/anxiety’ is significantly lower at the 2nd assessment than at the 1st assessment (Mdn. = 1 vs. Mdn. = 2; z = -3.500 *p* < 0.001, *r* = 0.48; *n* = 54). The intensity of ‘lack of drive’ is significantly lower at the 2nd assessment than at the 1st assessment (Mdn = 1 vs. Mdn. = 2; z = -3.482 *p* < 0.001, *r* = 0.47; *n* = 54). Also significantly lower is the intensity of ‘mood swings’ at the 2nd assessment compared to the 1st assessment (Mdn = 1 vs. Mdn. = 2; z = -3256 p = 0.001, r = 0.44; n = 54).

The intensity of the limitation in ‘carry out daily routine’ is the strongest change of ALPR between the first two assessments and is significantly lower at the 2nd assessment than at the 1st assessment (Mdn = 3 vs. Mdn. = 3; z = -3.181 *p* = 0.001, *r* = 0.43; *n* = 54). The data also show a significant reduction of limitations in ‘household’ (1st assessment Mdn. = 2, 2nd assessment Mdn. = 2, z = -2.616; *p* = 0.009; *n* = 54; *r* = 0.36) and ‘look after health’(1st assessment Mdn. = 2, 2nd assessment Mdn. = 1, z = -2.596; p = .009; n = 54; r = 0.35). The effect size r of the results is < 0.3 and corresponds to a medium effect.

### Subgroup analysis of women and men

Comparing means between women and men using Mann-Whitney-U-Test, it is noticeable that there are hardly any significant differences considering the intensity of the symptoms at the 1st assessment (exception: ‘restriction on movement’ and ‘hair loss’), but several significant differences regarding the ALPR. Women are more restricted in e.g.,‘household’ (Mdn = 2; U = 7283.000; *p* < 0.001; *r* = 0.2), ‘provide care for others’ (Mdn. = 3; U = 7225.000; *p* < 0.001; *r* = 0.2) and ‘get where you want to go‘ (Mdn. = 2; U = 7855.500; *p* = 0.009; *r* = 0.15) compared to men (‘household’ : Mdn. = 2; ‘care for others’ Mdn. = 2; ‘get where you want to go‘ Mdn. = 1). The effect size r is < 0.3 in each case and corresponds to a low effect. In the 2nd assessment the only significant difference between women and men remains in ‘get where you want to go‘ (Mdn = 2 (women) vs. 0 (men); U = 180.000; p = 0.015).

Examining the correlation of the intensity of each health problem with the intensity of each ALPR aspect using Spearman´s rank correlation, we found that 8 out of 20 health problems correlated significantly with all items of the ALPR assessment. For more details please see Table 2. This illustrates the correlation between the symptoms associated with COVID-19 and patients’ activities of daily living (ADL). The following are just a few examples: At the 1st assessment ‘fatigue’ correlates significantly with all ALPR aspects e.g., ‘carry out daily routines’, ‘household’ and ‘handle stress’. Further significant correlations can be found in, ‘concentration problems’ with all ALPR e.g., ‘handle stress’, ‘look after health’ and ‘interact with people’. More details and correlations are shown in Table 2.

At the 2nd assessment, we found fewer significant correlations, but more values corresponding to a strong effect.

**Table 2:** Spearman-correlation of health problems with limitations in activity and participation at 1st assessment.

*n* = 307	Carry out daily routines	Handle Stress	Use of hands and fingers	Get where you want to go	Use of public trans-portation	Use of private trans-portation	Look after health	House-hold	Provide care for others	Interact with people	Intimate relation-ships	Relaxation/ pleasure	Shortness of breath during physical exertion
Sleep disturbance													
r	0.182	0.267	0.234	0.252	0.198	0.108	0.245	0.220	0.214	0.157	0.230	0.278	0.234
p	0.001	< 0.001	< 0.001	< 0.001	< 0.001	0.058	< 0.001	< 0.001	< 0.001	0.006	< 0.001	< 0.001	< 0.001
Intestinal dysfunction													
r	0.130	0.086	0.249	0.241	0.186	0.164	0.160	0.223	0.196	0.145	0.230	0.179	0.173
p	0.023	0.133	< 0.001	< 0.001	0.001	0.004	0.005	< 0.001	< 0.001	0.011	< 0.001	0.002	0.002
Restrictions on movement													
r	0.155	0.084	0.481	0.209	0.164	0.168	0.134	0.234	0.216	0.170	0.215	0.149	0.213
p	0.006	0.144	< 0.001	< 0.001	0.004	0.003	0.019	< 0.001	< 0.001	0.003	< 0.001	0.009	< 0.001
Muscular problems													
r	0.382	0.160	0.394	0.322	0.241	0.145	0.253	0.385	0.318	0.125	0.202	0.235	0.299
p	< 0.001	0.005	< 0.001	< 0.001	< 0.001	0.011	< 0.001	< 0.001	< 0.001	0.028	< 0.001	< 0.001	< 0.001
Problems oft he respiratory tract													
r	0.286	0.105	0.213	0.222	0.147	0.023	0.278	0.266	0.212	0.165	0.177	0.164	0.690
p	< 0.001	0.067	< 0.001	< 0.001	0.010	0.682	< 0.001	< 0.001	< 0.001	0.004	0.002	0.004	< 0.001
Limitations of the sense of smell													
r	0.117	0.049	0.196	0.147	0.069	0.100	0.117	0.112	0.177	0.136	0.174	0.140	0.218
p	0.041	0.393	< 0.001	0.010	0.226	0.082	0.040	0.050	0.002	0.018	0.002	0.014	< 0.001
Limitations of the sense of taste													
r	0.118	0.042	0.220	0.113	− 0.001	0.120	0.108	0.139	0.144	0.101	0.194	0.139	0.183
p	0.039	0.461	< 0.001	0.048	0.984	0.035	0.058	0.015	0.011	0.077	< 0.001	0.015	0.001
Vascular occlusion													
r	0.117	0.035	0.109	0.143	0.176	0.084	0.081	0.126	0.181	0.101	0.142	0.141	0.046
p	0.040	0.538	0.056	0.012	0.002	0.140	0.156	0.027	0.001	0.076	0.013	0.014	0.423
Fatigue													
r	0.533	0.358	0.183	0.245	0.151	0.206	0.346	0.414	0.316	0.221	0.276	0.287	0.296
p	< 0.001	< 0.001	< 0.001	< 0.001	0.008	< 0.001	< 0.001	< 0.001	< 0.001	< 0.001	< 0.001	< 0.001	< 0.001
Hair loss													
r	0.126	0.140	0.164	0.182	0.194	0.075	0.146	0.195	0.192	0.097	0.160	0.113	0.084
p	0.028	0.014	0.004	0.001	< 0.001	0.190	0.011	< 0.001	< 0.001	0.090	0.005	0.049	0.141
Word-finding disorder													
r	0.199	0.264	0.233	0.194	0.163	0.182	0.288	0.253	0.235	0.362	0.297	0.201	0.232
p	< 0.001	< 0.001	< 0.001	< 0.001	0.004	0.001	0.001	< 0.001	< 0.001	< 0.001	< 0.001	< 0.001	< 0.001
Concentration problems													
r	0.355	0.393	0.162	0.235	0.174	0.237	0.371	0.298	0.254	0.353	0.355	0.290	0.338
p	< 0.001	< 0.001	0.004	< 0.001	0.002	< 0.001	< 0.001	< 0.001	< 0.001	< 0.001	< 0.001	< 0.001	< 0.001
Mood Swings													
r	0.252	0.412	0.159	0.246	0.213	0.083	0.222	0.220	0.211	0.312	0.367	0.393	0.215
p	< 0.001	< 0.001	0.005	< 0.001	< 0.001	0.146	< 0.001	< 0.001	< 0.001	< 0.001	< 0.001	< 0.001	< 0.001
Lack of drive													
r	0.345	0.390	0.113	0.193	0.111	0.085	0.294	0.282	0.200	0.329	0.317	0.381	0.287
p	< 0.001	< 0.001	0.047	< 0.001	0.052	0.138	< 0.001	< 0.001	< 0.001	< 0.001	< 0.001	< 0.001	< 0.001
Fear. anxiety													
r	0.247	0.445	0.052	0.279	0.248	0.152	0.226	0.262	0.252	0.321	0.310	0.389	0.187
p	< 0.001	< 0.001	0.363	< 0.001	< 0.001	0.008	< 0.001	< 0.001	< 0.001	< 0.001	< 0.001	< 0.001	0.001
Visual impairment													
r	0.168	0.103	0.250	0.287	0.245	0.239	0.269	0.285	0.263	0.207	0.268	0.206	0.198
p	0.003	0.072	< 0.001	< 0.001	< 0.001	< 0.001	< 0.001	< 0.001	< 0.001	< 0.001	< 0.001	< 0.001	< 0.001
Tinnitus													
r	0.182	0.191	0.200	0.223	0.225	0.230	0.243	0.243	0.210	0.193	0.231	0.221	0.170
p	0.001	< 0.001	< 0.001	< 0.001	< 0.001	< 0.001	< 0.001	< 0.001	< 0.001	< 0.001	< 0.001	< 0.001	0.003
Dizziness													
r	0.217	0.173	0.236	0.323	0.263	0.216	0.243	0.252	0.273	0.221	0.216	0.220	0.235
p	< 0.001	0.002	< 0.001	< 0.001	< 0.001	< 0.001	< 0.001	< 0.001	< 0.001	< 0.001	< 0.001	< 0.001	< 0.001
Tachycardia/ heart palpitations													
r	0.204	0.188	0.204	0.324	0.278	0.190	0.206	0.298	0.270	0.279	0.262	0.292	0.356
p	< 0.001	< 0.001	< 0.001	< 0.001	< 0.001	< 0.001	< 0.001	< 0.001	< 0.001	< 0.001	< 0.001	< 0.001	< 0.001
Pain in daily life													
r	0.316	0.182	0.337	0.337	0.226	0.180	0.239	0.342	0.315	0.215	0.275	0.297	0.293
p	< 0.001	0.001	< 0.001	< 0.001	< 0.001	0.002	< 0.001	< 0.001	< 0.001	< 0.001	< 0.001	< 0.001	< 0.001

Looking at the correlations separately for women and men, it is noticeable that different symptoms correlate with ALPR.

In women, at the 1st assessment ‘lack of drive’ correlates with 11 out of 13 ALPR aspects e.g., ‘handle stress’, ‘relaxation/pleasure’ and ‘carry out daily routines’ are significant. Further 12 out of 13 correlations between ‘muscular problems’ with ALPR e.g., ‘household’, ‘use of hands and fingers’ and ‘carry out daily routines’ are significant. In men, at the 1st assessment ‘problems of the respiratory tract’ correlations with 10 out of 13 ALPR aspects e.g. ‘shortness of breath during physical exertion’, ‘carry out daily routines’ and ‘look after health’ are significant. All correlations between ‘tachycardia/heart palpitations’ with ALPR e.g., ‘use of public transportation’, ‘carry out daily routines’ and ‘provide care for others’ are significant. Aside from that ‘fear/anxiety’ correlates with ‘handle stress’ with a strong effect.

At the 2nd assessment, in women we also find that ‘muscular problems’ correlate with 12 out of 13 ALPR aspects significantly, this time with higher effect sizes compared to the 1st assessment. In addition, there are significant correlations of ‘pain in daily life’ in 12 out of 13 ALPR aspects e.g., ‘use of hands and fingers’, ‘get where you want to go’ and ‘relaxation/pleasure’. In men, ‘problems of the respiratory tract’ and ‘tachycardia/heart palpitations’ correlate significantly in 8 vs. 9 out of 13 ALPR aspects, both this time with higher effect sizes compared to the 1st assessment. Table 3 shows details for the correlations (with highest effect sizes) of health problems with ALPR in women and men at different times of assessment ranked by effect sizes.

**Table 3:** Most prominent correlations of health problems with ALPR in women and men at different times of assessment ranked by effect sizes.

Health problems	ALPR
1st assessment; women; *n* = 219	
Lack of drive	1) Handle stress: *r* = 0.416; *p* < 0.001 2) Relaxation/pleasure: *r* = 0.387; *p* < 0.001 3) Carry out daily routines: *r* = 0.359; *p* < 0.001
Muscular problems	1) Household: *r* = 0.435; *p* < 0.001 2) Use of hands and fingers: *r* = 0.423; *p* < 0.001 3) Carry out daily routines: *r* = 0.384; *p* < 0.001
1st assessment; men; *n* = 88	
Problems of the respiratory tract	1) Shortness of breath during physical exertion: *r* = 0.740; *p* < 0.001 2) Carry out daily routines: *r* = 0.430; *p* < 0.001 3) Look after health: *r* = 0.442; *p* < 0.001
Tachycardia/heart palpitations	1) Use of public transportation: *r* = 0.486; *p* < 0.001 2) Carry out daily routines: *r* = 0.470; *p* < 0.001 3) Provide care for others: *r* = 0.452; *p* < 0.001
Fear/anxiety	1) Handle stress: *r* = 0.525; *p* < 0.001
2nd assessment; women; *n* = 38	
Muscular problems	1) Use of hands and fingers: *r* = 0.605; *p* < 0.001 2) Intimate relationships: *r* = 0.588; *p* < 0.001 3) Shortness of breath during physical exertion: *r* = 0.564; *p* < 0.001
Pain in daily life	1) Use of hands and fingers: *r* = 0.619. *p* < 0.001 2) Get where you want to go: *r* = 0.576; *p* < 0.001 3) Relaxation/pleasure: *r* = 0.509; *p* < 0.001
2nd assessment; men; *n* = 16	
Problems of the respiratory tract	1) Shortness of breath during physical exertion: *r* = 0.897; *p* < 0.001 2) Look after health: *r* = 0.819; *p* < 0.001 3) Household: *r* = 0.641; *p* = 0.008
Tachycardia/heart palpitations	1) Get where you want to go: *r* = 0.804; *p* < 0.001 2) Use of public transportation: *r* = 0.758; *p* < 0.001 3) Interact with people: *r* = 0.757; *p* < 0.001

Looking at the correlations based on the ALPR, the following stands out:

At the 1st assessment we find 20 out of 20 correlations between ‘carry out daily routines’ and health problems e.g., ‘fatigue’, ‘muscular problems’ and ‘concentration problems’ that are significant. ‘Handle stress’ correlates significantly with 12 out of 20 health problems e.g., ‘fear/anxiety’, ‘mood swings’ and ‘concentration problems’. ‘Interact with people’ correlates significantly with 17 out of 20 health problems e.g., ‘word-finding disorder’, ‘concentration problems’, and ‘lack of drive’. Further details can be found in Table 2. In the 2nd assessment ‘interact with people’ correlates significantly with 14 out of 20 health problems e.g., ‘concentration problems’, ‘fatigue’ and ‘tinnitus’. ‘Get where you want to go’ correlates significantly with 14 out of 20 health problems e.g., ‘pain in daily life’, ‘fatigue’ and ‘muscular problems’. ‘Use of private transportation’ correlates significantly with 12 out of 20 health problems e.g., ‘muscular problems’, ‘visual impairment’ and ‘dizziness’.

In women at the 1st assessment, we find fewer correlations regarding ALPR with different symptoms with a strong or medium effect than in men.

In men ‘provide care for others’ correlates significantly with 15 out of 20 health problems e.g., ‘tachycardia/heart palpitations’, ‘pain in daily life’ and ‘visual impairment. ‘Carry out daily routines’ correlates significantly with 9 out of 20 health problems e.g., ‘fatigue’, ‘tachycardia/heart palpitations’ and ‘problems of the respiratory tract’. ‘Relaxation/pleasure’ correlates significantly with 14 out of 20 health problems e.g., ‘mood swings’, ‘concentration problems’ and ‘tachycardia/heart palpitations’.

**Fig. 4:**
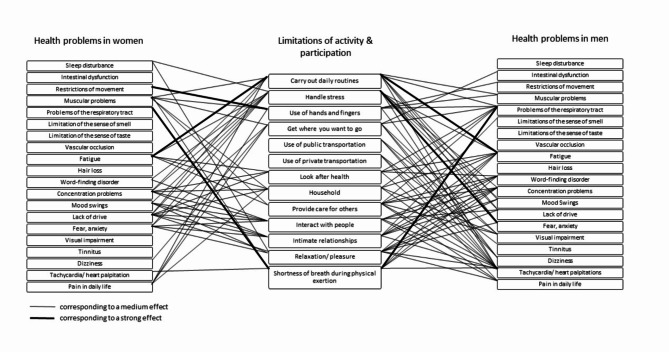
Correlations of health problems and ALPR in women and men with a strong or medium effect at the 1st assessment.

**Fig. 5:**
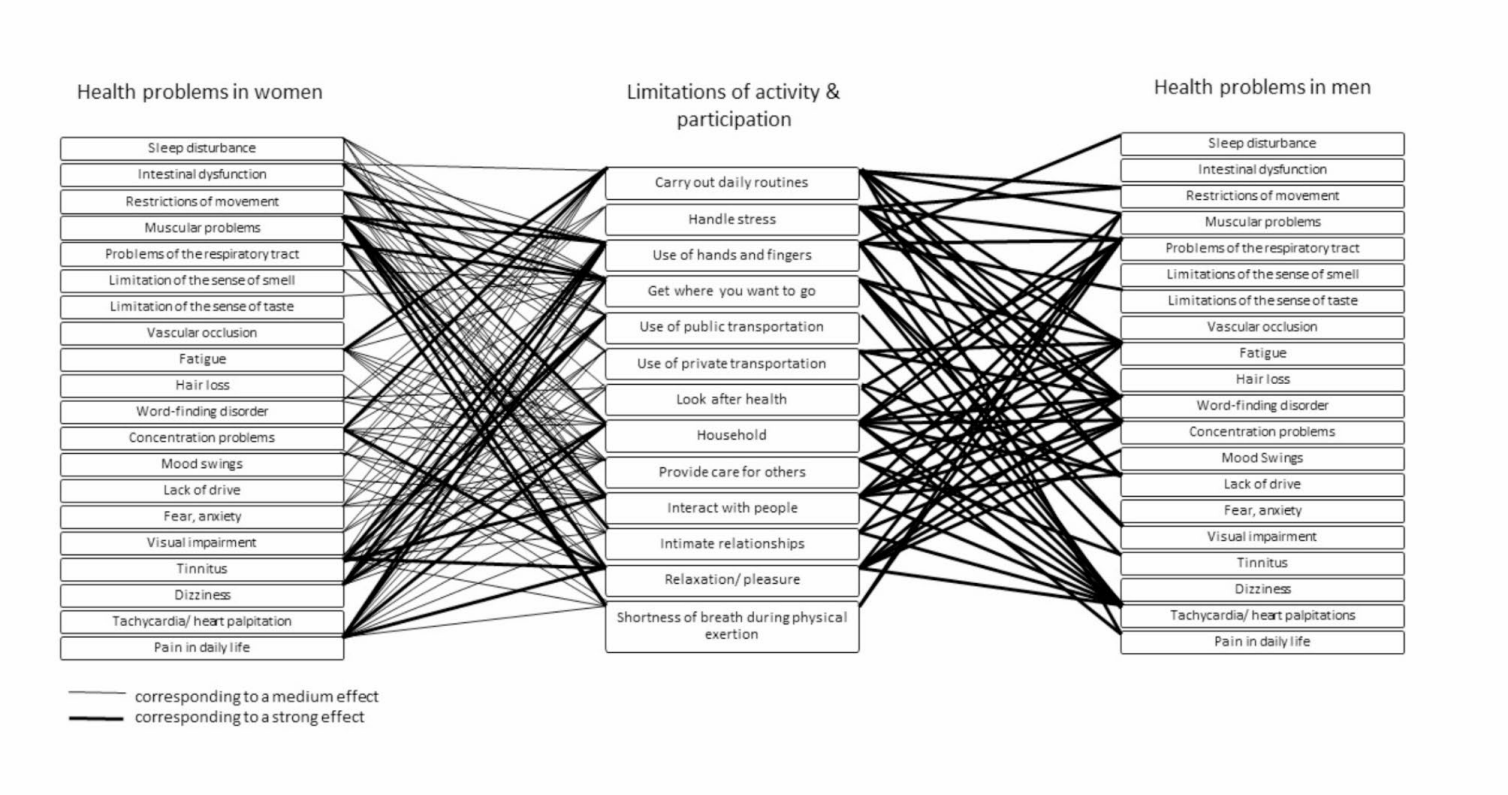
Correlations of health problems and ALPR in women and men with a strong or medium effect at the 2nd assessment.

In women at the 2nd assessment, 15 out of 20 correlations of ‘interact with people’ and 14 out of 20 correlations of ‘get where you want to go’ with health problems are significant. In men, we find fewer significant correlations, but the significant ones with higher effect sizes. More details are shown in Table 4. An overview of the correlations with a medium and strong effect can be found in Figs. 4 and 5.

**Table 4:** Most prominent correlations of ALPR with health problems of all study participants, in women and men, at different times of assessment, ranked by effect sizes.

ALPR	Health problems
2nd assessment, *n* = 54	
Interact with people	1) Concentration problems: *r* = 0.514; *p* < 0.001 2) Fatigue: *r* = 0.495; *p* < 0.001 3) Tinnitus: *r* = 0.484; *p* < 0.001
Get where you want to go	1) Pain: *r* = 0.517; *p* < 0.001 2) Fatigue: *r* = 0.483; *p* < 0.001 3) Muscular problems: *r* = 0.470; *p* < 0.001
Use of private transportation	1) Muscular problems: *r* = 0.405; *p* = 0.002 2) Visual impairment: *r* = 0.400; *p* = 0.003 3) Dizziness: *r* = 0.396; *p* = 0.003
1st assessment, men, *n* = 88	
Provide care for others	1) Tachycardia/heart palpitations: *r* = 0.452; *p* < 0.001 2) Pain in daily life: *r* = 0.432; *p* < 0.001 3) Visual impairment: *r* = 0.397; *p* < 0.001
Carry out daily routines	1) Fatigue: *r* = 0.529; *p* < 0.001 2) Tachycardia/heart palpitations: *r* = 0.470; *p* < 0.001 3) Problems of the respiratory tract: *r* = 0.430; *p* < 0.001
2nd assessment, women, *n* = 38	
Interact with people	1) Muscular problems: *r* = 0.568; *p* < 0.001 2) Tinnitus: *r* = 0.539; *p* < 0.001 3) Pain in daily life: *r* = 0.486; *p* = 0.001
Get where you want to go	1) Pain in daily life: *r* = 0.576; *p* < 0.001 2) Problems of the respiratory tract: *r* = 0.513; *p* < 0.001 3) Muscular problems: *r* = 0.506; *p* = 0.001

## Discussion

In this study we found that fatigue is one of the most common symptoms (92.8% (*n* = 285) at the 1st assessment) of the study participants with post-COVID symptoms. This aligns with the findings of other studies which report fatigue as the most common symptom with an incidence around 85%^1,13,14^. Fatigue after COVID-19 infection is likely to be one of the symptoms most related with functional status^15^, this is consistent with our findings, which show frequent correlations between fatigue and ALPR.

There is little data on the impact on the functional status of Post-COVID-Condition (PCC)^9^, although it is suggested that rehabilitation should be pursued in a holistic approach following the International Classification of Functioning, Disability and Health (ICF)^16^.

Our data show correlations, partly with a large effect size, between ‘fatigue’ or ‘concentration problems’ and ‘carry out daily routines’.

In their study, Lemhöfer et al. reported that 49% of the study participants indicate long-term ALPR after more than 3 months, most commonly ‘handle stress’, ‘carry out daily routines’ and ‘shortness of breath during physical exertion’^9^. This matches our results regarding the most frequent ALPR. Fernández-de-las-Peñas et al. found in a multicenter study that 20 − 30% of hospitalized COVID-19-patients reported limitations in ADL 8 months after the hospitalization. The functional limitations were categorized in occupational activities, leisure/social activities, basic activities, and instrumental activities^17^. This is consistent with the results of a Danish descriptive cross-sectional study, which found a lower level of functional status in patients with post-COVID compared to their functional status prior infection with COVID-19, using post-COVID-19 functional status scale (PCFS). They also reported, that 94% of the patients indicate slight to moderate functional limitations and that only 5% were classified as having no or negligible functional limitations at the time of assessment, whereas 94% were categorized as such at the time before infection^18^. In contrast to that an Indian study, using PCFS, found that 87% of their participants did not report functional limitations in ADLs, only 2% reported reduced ability and 0,3% required constant assistance in their daily activities, compared to their state pre-COVID^19^. The PCFS uses a global score and does not test which ADL is limited^17^. It is designed as an additional outcome measure to analyze effects of COVID-19 on functional status over time^20^. In a cross-sectional study Fugazzaro et al. investigated the outcomes of participants 3 months after hospital discharge for COVID-19 regarding ADL and participation. They reported that 91,3% of the study participants claimed complete independence in B-ADL, while 76% did not fully reintegrated in participation, which might be related to ongoing impact of disease or continued shut down of social facilities, as the authors discussed^21^.

Other recent studies focus on changes in quality of life rather than individual ADL: In their meta-analysis, Malik et al. found poor quality of life in 58% of post-COVID patients, which matches our results using the SF-36 questionnaire, and using pooled analysis in the EQ-5Q-5 L questionnaire, that 28% had challenges with usual activities^22^. Study participants are likely to answer item ‘usual activities’ in the EQ-5Q-5 L questionnaire similar to the item ‘carry out daily routines’ in the RehabNeQ which was used in this study. This could lead to a comparison of the results of the items, whereas the restrictions found in this study are considerably higher (69.7%, n = 214). The poor assessment of quality of life might not only be attributable to post-COVID symptoms, but other pandemic-related aspects such as fewer social contacts and insecurities regarding employment might be of importance^13,23^, as well as the impact of the lockdown on lifestyle of the population in general such as diet and physical activity^24^.

Supporting the results of above mentioned studies, our findings indicate a need for rehabilitation: Given the many different symptoms of PCC and many possible comorbidities, many authors suggest a multidisciplinary, multimodal and individualized approach to rehabilitation^25–27^, which has shown to be effective in the post-acute phase of COVID-19^28^. In addition, rehabilitative care during and immediately after the infection is important. A study examining this topic found, that 22% of study participant said that they had difficulty getting therapy during the infection. Further, many respondents wanted the opportunity to present themselves in a COVID-19 aftercare facility^29^. To date, there are no high-quality trials on rehabilitation of PCC, so it is initially necessary to rely on expert opinions, which pose the risk of bias or conflict of interests^30^ and high-quality research regarding rehabilitation methods, which proved to be effective for similar symptoms but in health contexts other than PCC. This kind of research is done, for example, with the article series “Cochrane ‘evidence relevant to’ rehabilitation of people with post COVID-19 condition“^31^.

Exercise rehabilitation could lead to improvements of physical and psychological symptoms, but exact details such as the timing of rehabilitation have not yet been clarified and require further research^32^. At the same time, the WHO identified post-exertional symptom exacerbation as a red flag for rehabilitation in PCC-patients^33^.

Further, it should be considered regarding symptoms and ALPR occurring after a COVID-19 infection, that it is not possible to distinguish whether these health problems are due only to the COVID-19 infection or whether they are influenced by pre-existing comorbidities.

In addition to treating symptoms, we suggest short-term relief options that support patients in their limited activities, such as help with household chores and care for children or relatives. Relaxation or stress management programs are also conceivable.

Our data show a significant change in the intensity of the health problem ‘fear/anxiety’ and ALPR ‘carry out daily routine’ over time. However, the median did not change over time for both items, which leads to the question, whether this change is clinically relevant.

Considerably more women than men presented in this study at the 1st assessment and the 2nd assessment. Reason for this could be that PCC seems to occur more often in women than in men^34^, as female sex is a risk factor for long-term symptoms^7,15,35,36^. In addition, men may generally seek medical help later due to some social norms^37,38^. We found almost no significant difference in symptom intensity in female and male participants. These results differ from findings of other studies. Huang et al. observed that women are more likely to experience fatigue, muscle weakness, anxiety or depression, and lung diffusion impairment compared to men^39^ Paradowska-Nowakowska et al. found many symptoms significantly more frequent in women e.g., myalgia, palpitation, concentration disorders and depressed mood^34^.

While the differences in symptom intensity between women and men in our study did not differ significantly, the intensity of ALPR did. Women experience more restrictions in activities such as ‘household’ and ‘provide care for others’, which could also be since they carry out these tasks more often as part of a classic distribution of roles. Of particular interest are the different correlations depending on sex between the intensity of the symptoms and ALPR. At the 1st assessment, in women we found many correlations of the intensity of ‘muscular problems’ or ‘lack of drive’ or ‘anxiety’ with the intensity of ALPR, while for men ‘problems of the respiratory tract’ or ‘tachycardia/palpitation’ correlated with experienced limitations in ADLs.

At the 2nd assessment we found notably more correlations with stronger effect sizes.

This may be because participants with a greater burden of disease are more likely to present again. Women seem to experience mainly ALPR in combination with ‘restrictions of movement’, ‘muscular problems’, and ‘pain in daily life’, whereas in men this was the case for ‘problems of the respiratory tract’ and ‘tachycardia/heart palpitations’.

These correlations suggest that women and men experience different restrictions in their daily activities in combination with different symptoms. This could also lead to different rehabilitation needs for women and men. However, further research is required to confirm these assumptions.

The strength of this study is the consideration of the ALPR and the correlations with the intensities of the individual symptoms. This provides an indication of how the study participants’ health problems affect their everyday lives and what rehabilitation needs arise as a result. This is an important note for further studies, as our data only show correlations and not causal relationships. Furthermore, this study suggests that women and men experience different ALPR and therefore probably have different needs for rehabilitation. Despite this, the results of this study must be considered with several limitations. First, the high loss-to-follow-up should be mentioned, the reasons for this can only be assumed. Retrospectively, the medical team of the university outpatient clinic of the Department of Rehabilitation- and Sports Medicine at Hannover Medical School reflects on the following reasons: for many patients, the doctors primarily take on an advisory role, after the 1st assessment the needs of some participants are met, e.g., explanations of the interrelationships and tips for your own treatment and handling of the symptoms. Further study participants travel long distances to attend consultation hours, as there are only a few specialist outpatient clinics for post-COVID patients in Germany. This could affect the possibility of another appointment. In addition, appointments often cannot be arranged in a timely manner due to capacity reasons, so that some participants present again after a year and therefore the data of the re-assessment is not included in our evaluation period. The loss-to-follow-up makes it difficult to monitor and evaluate the development of health problems and ALPR over time. Aside from that, the health problems were not clinically objectified but are based on the self-assessment of the study participants. There is also no information about e.g., treatments of symptoms, re-infection with Sars-CoV-19 or other factors in the period between assessments, which could affect the participants’ outcome. Comorbidities that already existed before the COVID-19 infection were also not included in the analysis of the study data, although they may have an influence on the outcome of the individual participants. A selection bias cannot be ruled out because only participants who attended a consultation appointment at the special outpatient clinic for post-COVID patients were included in the study. The participants did not need a confirmed PCR result of their COVID-19 infection, which is another limitation.

In conclusion, this study found that study participants with post-COVID symptoms also experience ALPR. Moreover, we noticed frequent correlations between fatigue, as one of the most common health problems, and several ALPR. While these correlations apply to both sexes, we also found different correlations between health problems and ALPR in women and men. In women we found many correlations of ‘lack of drive’ or ‘muscular problems’ or ‘pain in daily life’ with different ALRP, whereas for men this was the case for ‘problems of the respiratory tract’ and ‘tachycardia/heart palpitations’.

## Data Availability

The data presented in this study are available on request from the corresponding author. The data are not publicly available due to a data protection act.
